# Sex-Specific Effects of Nanoparticle-Encapsulated MitoQ (nMitoQ) Delivery to the Placenta in a Rat Model of Fetal Hypoxia

**DOI:** 10.3389/fphys.2019.00562

**Published:** 2019-05-24

**Authors:** Esha Ganguly, Mais M. Aljunaidy, Raven Kirschenman, Floor Spaans, Jude S. Morton, Thomas E. J. Phillips, C. Patrick Case, Christy-Lynn M. Cooke, Sandra T. Davidge

**Affiliations:** ^1^ Department of Physiology, University of Alberta, Edmonton, AB, Canada; ^2^ Department of Obstetrics and Gynaecology, University of Alberta, Edmonton, AB, Canada; ^3^ Women and Children’s Health Research Institute, University of Alberta, Edmonton, AB, Canada; ^4^ Dementia Research Institute, Cardiff University, Cardiff, United Kingdom; ^5^ Musculoskeletal Research Unit, University of Bristol, Bristol, United Kingdom

**Keywords:** placenta, hypoxia, MitoQ, nanoparticles, sex difference

## Abstract

Pregnancy complications associated with chronic fetal hypoxia have been linked to the development of adult cardiovascular disease in the offspring. Prenatal hypoxia has been shown to increase placental oxidative stress and impair placental function in a sex-specific manner, thereby affecting fetal development. As oxidative stress is central to placental dysfunction, we developed a placenta-targeted treatment strategy using the antioxidant MitoQ encapsulated into nanoparticles (nMitoQ) to reduce placental oxidative/nitrosative stress and improve placental function without direct drug exposure to the fetus in order to avoid off-target effects during development. We hypothesized that, in a rat model of prenatal hypoxia, nMitoQ prevents hypoxia-induced placental oxidative/nitrosative stress, promotes angiogenesis, improves placental morphology, and ultimately improves fetal oxygenation. Additionally, we assessed whether there were sex differences in the effectiveness of nMitoQ treatment. Pregnant rats were intravenously injected with saline or nMitoQ (100 μl of 125 μM) on gestational day (GD) 15 and exposed to either normoxia (21% O_2_) or hypoxia (11% O_2_) from GD15 to 21. On GD21, placentae from both sexes were collected for detection of superoxide, nitrotyrosine, nitric oxide, CD31 (endothelial cell marker), and fetal blood spaces, *Vegfa* and *Igf2* mRNA expression in the placental labyrinth zone. Prenatal hypoxia decreased male fetal weight, which was not changed by nMitoQ treatment; however, placental efficiency (fetal/placental weight ratio) decreased by hypoxia and was increased by nMitoQ in both males and females. nMitoQ treatment reduced the prenatal hypoxia-induced increase in placental superoxide levels in both male and female placentae but improved oxygenation in only female placentae. Nitrotyrosine levels were increased in hypoxic female placentae and were reduced by nMitoQ. Prenatal hypoxia reduced placental *Vegfa* and *Igf2* expression in both sexes, while nMitoQ increased *Vegfa* and *Igf2* expression only in hypoxic female placentae. In summary, our study suggests that nMitoQ treatment could be pursued as a potential preventative strategy against placental oxidative stress and programming of adult cardiovascular disease in offspring exposed to hypoxia *in utero*. However, sex differences need to be taken into account when developing therapeutic strategies to improve fetal development in complicated pregnancies, as nMitoQ treatment was more effective in placentae from females than males.

## Introduction

Chronic fetal hypoxia, a common consequence of pregnancy complications (e.g., placental insufficiency), has been linked to the development of cardiovascular and metabolic diseases in the adult offspring. The placenta serves as feto-maternal interface for the exchange of nutrients and oxygen to the fetus. Placental insufficiency is often associated with placental oxidative and nitrosative stress (i.e., formation of reactive oxygen/nitrogen species; ROS/RNS). An imbalance in ROS/RNS levels caused by excessive generation of ROS and/or a fall in endogenous antioxidants such as superoxide dismutase (SOD) can lead to impaired placental development and altered placental function (reviewed in Al-Gubory et al., 2010; Schoots et al., 2018). One impact of excessive superoxide anions is the resultant scavenging of nitric oxide (NO) to produce RNS (e.g., peroxynitrite; Myatt and Cui, 2004); therefore, increased superoxide levels could reduce NO bioavailability and impair the important contribution of NO in feto-placental angiogenesis (Webster et al., 2008).

Interestingly, the placental response to oxidative stress appears to be different in placentae from males compared with females (reviewed in Rosenfeld, 2015). Human studies have shown that the placental oxidative stress response to adverse maternal environments (such as oxidative/nitrosative stress and reduced levels of antioxidants) in general appears to be more pronounced in male versus female placentae (Stark et al., 2011; Sedlmeier et al., 2014; Muralimanoharan et al., 2015; Evans and Myatt, 2017). In rodents, similar results were found, where adverse maternal environments (such as prenatal hypoxia) altered placental morphology, placental gene expression, and enzymes for epigenetic modifications (e.g., DNA methylation) in a sexually dimorphic manner (Mao et al., 2010; Gabory et al., 2012; Kim et al., 2014; Thompson et al., 2018). Therefore, examining sex-specific differences in the placental responses to adverse maternal environments or placental phenotypes as an outcome of the adverse environments is warranted.

As the placenta lacks both autonomic and cholinergic innervation, placental morphology and function are dependent on locally derived growth factors such as vascular endothelial growth factor A (VEGFA) and insulin-like growth factor 2 (IGF2; Reynolds and Redmer, 2001; Marzioni et al., 2004). Expression of the pro-angiogenic factor VEGFA is regulated by the oxygen sensing family of transcription factors such as hypoxia-inducible factor-1 alpha (HIF-1α; Cuffe et al., 2014). Placental hypoxia has been shown to impair feto-placental vascular development *via* VEGFA and HIF-1α (Kulandavelu et al., 2013). Decreased IGF2 expression is also associated with intrauterine growth restriction (IUGR) and critical to placental morphology and nutrient transfer to the fetus (Coan et al., 2008). In addition, hypoxia and placental oxidative/nitrosative stress may cause mitochondrial dysfunction, DNA damage, or reduced expression of the antioxidant system, all of which can affect normal placental function and potentially reduce oxygen and nutrient delivery, thus impairing fetal development (Droge, 2002; Lappas et al., 2010; Thompson and Al-Hasan, 2012). Therefore, given that oxidative stress is a central mediator of placental dysfunction in fetal hypoxia, prenatal treatment of placental oxidative stress could potentially prevent the long-term effects of fetal hypoxia on adult offspring.

Mitochondria are a major source of cellular ROS (as reviewed in Holland et al., 2017), and human placental tissues from complicated pregnancies were shown to have increased mitochondrial content, decreased respiratory chain complex expression, and impaired mitochondrial function (as reviewed in Mando et al., 2014; Chiaratti et al., 2015; McCarthy and Kenny, 2016). Therefore, our lab and others have been studying the potential use of the mitochondrial-targeted antioxidant MitoQ to target placental oxidative stress in complicated pregnancies (Phillips et al., 2017; Aljunaidy et al., 2018; Nuzzo et al., 2018). MitoQ consists of a ubiquinone moiety linked to a positively charged lipophilic cation allowing for accumulation on the inner mitochondrial membrane, making it highly effective in preventing mitochondrial oxidative stress (Smith et al., 1999; Ross et al., 2005; Murphy and Smith, 2007). Indeed, in a rat model of prenatal hypoxia, MitoQ treatment prevented the increase in mitochondrial stress markers in the placental labyrinth zone (Nuzzo et al., 2018). Recently, we have shown that MitoQ encapsulated into polymeric nanoparticles (nMitoQ) is a delivery approach to access the placental syncytium without crossing the placental basal membrane to reach the fetus (Aljunaidy et al., 2017; Phillips et al., 2017). With this treatment strategy, we showed that maternal treatment with nMitoQ in a rat model of prenatal hypoxia prevented placental oxidative stress, increased fetal weight in female fetuses, improved neuronal development, and had sex-dependent beneficial effects on *in vivo* cardiovascular function in prenatally hypoxic adult offspring (Phillips et al., 2017; Aljunaidy et al., 2018). Furthermore, nMitoQ treatment of human preeclamptic placental explants improved neuronal development *in vitro* (Scott et al., 2018). However, the effect of nMitoQ treatment on placental morphology, the mechanisms *via* which reduced placental oxidative stress might improve placental function and fetal growth, and any potential sex differences are still under investigation.

In the current study, we aimed to further identify the effect of nMitoQ treatment on placental function and oxidative stress, and the sex-specific effects of the treatment, in a rat model of prenatal hypoxia. We hypothesized that antioxidant nMitoQ treatment would decrease prenatal hypoxia-induced oxidative/nitrosative stress along with increasing VEGFA expression, improving placental morphology, increasing IGF2 expression, and ultimately resulting in improved fetal growth. Moreover, since it has been shown that placentae from male and female fetuses respond to prenatal stress differently, we hypothesized that there is a sex-specific divergence in the placental response to prenatal hypoxia-induced oxidative/nitrosative stress and the effectiveness of nMitoQ treatment.

## Materials and Methods

### Ethics Approval

All procedures described were approved by the University of Alberta Animal Policy and Welfare Committee and were in accordance with the guidelines of the Canadian Council on Animal Care (AUP #242).

### Preparation of Nanoparticle Encapsulated MitoQ (nMitoQ)

MitoQ loaded nanoparticles were synthesized as previously described (Phillips et al., 2017; Aljunaidy et al., 2018). Briefly, an amphiphilic copolymer of poly (γ-glutamic acid) and L-phenylalanine ethyl ester (γ-PGA-Phe) was synthesized as described previously (Kim et al., 2009). γ-PGA-Phe (10 mg/ml) dissolved in dimethyl sulfoxide (DMSO) was added to an equivalent volume of sodium chloride (NaCl; 0.15 M), dialyzed against distilled water using a dialysis membrane, freeze-dried, and resuspended in phosphate-buffered Saline (PBS; 10 mg/ml). Nanoparticles were then measured by dynamic light scattering (Zetasizer Nano ZS, Malvern Instruments, UK) as diameter (180 nm), zeta potential (−20 mV), and polydispersity index (0.12). γ-PGA-Phe nanoparticles (10 mg/ml) were mixed with MitoQ (2 mg/ml) at equivalent volume in NaCl (0.2 M). Nanoparticles were isolated by centrifugation, washed, and resuspended in PBS (10 mg/ml). The amount of MitoQ (278 nm), which was adsorbed to nanoparticles, was evaluated by UV absorption measurement, as previously described (Phillips et al., 2017).

### Prenatal Hypoxia Rat Model

Female Sprague-Dawley rats, 3 months of age (weighing 250–275 g), were obtained from Charles River (Quebec, Canada) and housed in a temperature and light controlled room (10:14 h light/dark cycle) with *ad libitum* access to food and water. Females were housed with Sprague-Dawley males overnight, and pregnancy was confirmed the following morning by the presence of sperm in a vaginal smear, which was defined as gestational day (GD) 0 of pregnancy. On GD15, pregnant dams were randomly assigned into two groups that were exposed to either prenatal hypoxia (11% O_2_) by placing them in a Plexiglas hypoxic chamber from GD15 to 21 or were housed at atmospheric oxygen (21% O_2_) as controls. Pregnant dams received an intravenous injection *via* the tail vein on GD15 with either saline or nMitoQ (100 μl of 125 μM nMitoQ). As nMitoQ is recycled and lasts up to 1 week *in vivo*, the nMitoQ treatment protocol consisted only of a single injection (Phillips et al., 2017). The dose of nMitoQ was based on our previous studies (Phillips et al., 2017; Aljunaidy et al., 2018). As our study is focused on nMitoQ, as a single entity, we have a saline control rather than a nanoparticle along group as the properties may be different without the MitoQ and, ultimately, nanoparticles alone would never be used in practice. Previous studies have demonstrated that these nanoparticles are inert (Phillips et al., 2017; Scott et al., 2018). At the end of gestation, on GD21, rats were euthanized prior to parturition and fetal and placental weights were measured. Whole placentae (labyrinth and junctional zone) from male and female fetuses (two/sex/litter) were processed and embedded in either paraffin or optimal cutting temperature (OCT) compound for immunofluorescent and other staining procedures, as listed below. In other placentae (two/sex/litter), the placental labyrinth zones were isolated and snap frozen in liquid nitrogen for RNA and DNA methylation analysis.

### Dihydroethidium Staining for Superoxide Production and Diaminofluorescein-FM (DAF-FM) for Nitric Oxide Levels

Placental cryostat sections (10 μm) were thawed, washed three times with Hank’s balanced salt solution (HBSS), and incubated with dihydroethidium (DHE) to measure superoxide levels (200 μM, Biotium, Burlington, Canada) or DAF-FM to measure nitric oxide levels (10 μM, Thermo Fisher Scientific, Eugene, OR, USA) in HBSS at 37°C for 30 min. Sections were washed with HBSS (3 × 2 min) and covered with a drop of 4′,6-diamidino-2-phenylindole (DAPI) (Vector Laboratories). Sections were protected from light, and pictures were immediately taken to prevent photobleaching.

### Immunofluorescent Nitrotyrosine Staining for Placental Peroxynitrite Levels and CD31 Staining to Assess Placental Labyrinth Feto-Placental Vascular Capillaries

Nitrotyrosine residues are the molecular footprint of peroxynitrite generation and can be used as a marker of peroxynitrite (Webster et al., 2008). CD31 was used as an endothelial marker (Newman, 1997) to assess placental vascularization. Placental cryostat sections (8 μm) were thawed, fixed in ice-cold acetone for 10 min, washed thrice in PBS, and non-specific binding was blocked using 2% bovine serum albumin (BSA) in PBS for 60 min. Sections were incubated overnight at 4°C with a primary antibody for nitrotyrosine (1:10; mouse-anti-tyrosine, NOVUS Biologicals, Oakville, ON, Canada) or CD31 (1:200 mouse-anti-CD31/PECAM-1, NOVUS Biologicals) in 2% BSA/PBS. The next day, sections were washed thrice with PBS and incubated with secondary antibody [1:250 in 2% BSA/PBS; donkey anti-mouse IgG (H + L), AF488, Alexa, Invitrogen] for 1 h at room temperature. Sections were washed with PBS thrice, mounting medium containing DAPI was added (Vector Laboratories; Burlingame, CA, U.S.A) and slides were covered, protected from light and left to dry overnight. Images were taken the next day.

### Immunofluorescent Staining for Placental HIF-1α Expression

Placental levels of the transcription factor hypoxia-inducible factor-1α (HIF-1α) were measured as a marker of hypoxia. Placental PFA-fixed sections (8 μm) were dewaxed in xylene and rehydrated in ethanol (100, 95 and 80%). Endogenous peroxidase activity was blocked using 10% H_2_O_2_ in distilled water for 10 min and incubated in sodium citrate buffer supplemented with 0.05% Tween 20 at 90°C for 20 min for antigen retrieval. Non-specific staining was blocked with 2% BSA/PBS for 60 min at room temperature and incubated overnight at 4°C with a primary antibody against HIF-1α (1:250; rabbit-anti-HIF1 alpha, NOVUS Biologicals, Oakville, ON, Canada) in 2% BSA/PBS. The next day, sections were washed twice with Tris buffered saline containing 0.05% Tween 20 (TBS-T) and incubated with secondary antibody [1:250; goat-anti-rabbit IgG (H + L)-AF546, Invitrogen, Carlsbad, CA, USA] in TBS-T for 60 min at room temperature. After incubation with secondary antibody, sections were washed twice in TBS-T, once in distilled water and mounted using mounting medium containing DAPI (Vector Laboratories). Slides were protected from light and left to dry overnight. Images were taken the next day.

### Immunofluorescent Staining for Placental and Fetal Tissue Oxygenation

In a separate group of dams, tissue oxygenation levels were assessed by intraperitoneal (i.p.) injection of either pimonidazole hydrochloride (60 mg/kg) (Hypoxyprobe™-1, Burlington, USA) or an equivalent volume of vehicle (saline) as a control on GD20. Six hours post injection, dams were euthanized, and placentae, fetal hearts, and fetal liver were collected and snap frozen. Pimonidazole levels in placental tissues and fetal cardiac and hepatic tissues from both sexes were assessed by immunostaining. Pimonidazole hydrochloride (also known as 2-nitroimidazoles) distributes to all tissues but is activated by reduction in cells exposed to oxygen concentration less than 14 micromolar, which is equivalent to a partial pressure pO_2_ = 10 mmHg at 37°C. The activated intermediate forms stable adducts with proteins containing thiol groups (i.e., reduced pimonidazole, the staining product). Placental cryosections (8 μm) were fixed in acetone (10 min) and washed in PBS thrice, and non-specific staining was blocked using 2% BSA/PBS for 1 h. Sections were incubated overnight at 4°C with monoclonal anti-pimonidazole antibody (1:200; Hypoxyprobe™ Kit) in 2% BSA/PBS. The next day, sections were washed thrice in PBS and incubated with secondary antibody [1:250; donkey anti-mouse IgG (H + L), AF488, Alexa, Invitrogen] in 2% BSA/PBS for 60 min. Sections were washed with PBS three times and mounted using mounting medium containing DAPI (Vector Laboratories). Slides were protected from light and left to dry overnight. Images were taken the next day.

### Morphological Analysis of Placenta

Using an established hematoxylin and eosin (H&E) staining protocol, placental PFA-fixed sections (8 μm) were dewaxed in histoclear, rehydrated, stained with filtered Harris’s hematoxylin for 3 min, washed with distilled water, and then put into filtered eosin for 30 s. The placental sections were washed in cold water, covered, and left to dry overnight.

### Image Analysis

All images were taken on an Olympus IX81 fluorescence microscope with CellSens Dimensions software (Olympus, Japan) with TRITC at 532 nm (for DHE, HIF-1α staining) or FITC at 488 nm (for nitrotyrosine, DAF-FM and CD31 staining) wavelength, respectively. Three representative pictures of the placental labyrinth zone were randomly taken from each of the tissue section at 20× magnification. All pictures were corrected to the blank controls (i.e., samples without DHE, or samples incubated only with secondary antibodies) to remove background staining. Fluorescent images were analyzed using ImageJ 1.48 software (National Institutes of Health, Bethesda, MD, USA) to determine mean fluorescence intensity (MFI). MFI values from the DHE, nitrotyrosine, and pimonidazole staining were normalized to the nuclei counts per image. The average MFI of the three representative images per experimental group was taken.

For placental morphological analysis, images were taken with a digital camera mounted on a bright field microscope (EVOS XL Core Imaging System, Thermo Fisher Scientific, Canada) at a 2× magnification. For the other assessments, three randomly selected representative fields from each placenta were obtained at a magnification of 40×. Then, using ImageJ software, placental blood space area in each field of view was converted into black and remaining placental tissue into white for quantification. Total area of fetal blood space per field of view was calculated using ImageJ software, and the values were averaged per experimental group. Briefly, images were opened in ImageJ and converted into 16 bit binary images. Following which a threshold was set automatically by the program, which converted the placental blood space area into black and remaining placental tissue into white. Particle count of placental blood space was analyzed on ImageJ using the option of “analyze particles” in the software, which resulted in a surface area value for the black space, i.e., the placental blood space.

### Real-Time RT-PCR for Placental Gene Expression of IGF and VEGFA

Total RNA was isolated from the placental labyrinth using RNeasy plus Mini Kit (QIAGEN Inc., Ontario, Canada). Total RNA was reverse transcribed to cDNA using the High Capacity cDNA Reverse Transcription Kit (AB Applied Biosystems, Warrington, UK) according to the manufacturer’s instructions. Using gene-specific primers for *Igf2, Igf2P0, Igf1r, Igf2r,* and *Vegfa,* quantitative real time RT-PCR (qPCR) was performed using iQ™ SYBR Green Supermix (Bio-Rad, Hercules, CA, USA; see Table 1 for primers). Briefly, thermal cycling was initiated by a 5 min. Denaturation at 95°C, followed by 40 cycles of 95°C for 30 s, annealing at 60°C for 15 s, and 72°C for 30 s. Samples without reverse transcriptase (RT) using the same PCR primers were done as a control for the presence of genomic DNA. The gene expression levels in each sample (absolute quantification) were calculated from the standard curve (for each primer set) and normalized to rat Cyclophilin A (PPIA) expression.

**Table 1:** Quantitative real-time PCR primers.

Gene	Forward primer (5′–3′)	Reverse primer (5′–3′)
*Igf2*	TGT CTA CCT CTC AGG CCG TAC TT	TCC AGG TGT CGA ATT TGA AGA A
*Igf2P0*	GAT CAT CGT CCA GGC AAT TT	GTT GCG TAG TTC CCG AAG TT
*Igf1r*	AAG GAT GGC GTC TTC ACC A	GAG TGG CGA TCT CCC AGA G
*Igf2r*	CTG CAG GCG GGA AAG	TTC CAC TCT TAT CCA CAG CAC
*Vegfa*	GTG CAC TGG ACC CTG GCT TT	TTC ACC ACT TCA TGG GCT TTC TG
*Ppia*	AGC ATA CAG GTC CTG GCA TC	TTC ACC TTC CCA AAG ACC AC

### Statistical Analysis

Statistical analyses were performed using GraphPad Prism 7.04 software (GraphPad Software, U.S.A.). All data are expressed as mean ± SEM. All data were analyzed using a two-way ANOVA, with hypoxia and nMitoQ treatment as the two independent factors, followed by Sidak’s multiple comparison *post hoc* tests. A value of *p* < 0.05 was considered significant.

## Results

### Offspring and Placental Characteristics

In male fetuses, prenatal hypoxia decreased fetal weight and abdominal girth without affecting placental weight (Table 2). nMitoQ treatment increased male abdominal girth in the prenatal hypoxia exposed group, while no effects of nMitoQ treatment were observed on fetal or placental weights in males (Table 2). Prenatal hypoxia reduced placental efficiency and expressed as ratio of fetal weight/placental weight in male fetuses, which was not significantly improved by nMitoQ treatment; however, placental efficiency in the hypoxia/nMitoQ group was no longer significantly different than normoxia/saline controls (Table 2), suggesting an effect of nMitoQ treatment. Crown-to-rump length was similar between all experimental groups in male fetuses (Table 2).

**Table 2:** Fetal and placental characteristics of male offspring.

Variables	Normoxia		p-Hypoxia		Main effect		
	Saline	nMitoQ	Saline	nMitoQ	p-Hypoxia	nMitoQ	Interaction
Fetal weight (g)	5.81 ± 0.24	5.6 ± 0.15	5.21 ± 0.19	5.13 ± 0.26	*	–	–
Placental weight (g)	0.61 ± 0.04	0.58 ± 0.03	0.61 ± 0.02	0.62 ± 0.03	–	–	–
Fetal weight/placental weight	9.05 ± 0.39	9.76 ± 0.50	7.96 ± 0.78	8.69 ± 0.56	–	–	–
Abdominal girth (cm)	3.87 ± 0.08	3.72 ± 0.05	3.32 ± 0.13	**3.82** ± **0.13** ^†^	*	–	**
Crown-rump length (cm)	4.78 ± 0.09	4.92 ± 0.12	4.47 ± 0.18	4.82 ± 0.13	–	–	–

Body weight, placental weight, placental efficiency (body weight/placental weight), abdominal girth, and crown-rump length from male fetuses collected on GD21. Data are represented as mean ± SEM. All groups were compared using a two-way ANOVA followed by Sidak’s *post hoc* test.

* *p* < 0.05 overall group effect of prenatal hypoxia.

** *p* < 0.01 interaction between prenatal hypoxia and nMitoQ treatment.

† *p* < 0.05 versus corresponding hypoxia-saline group. Exact p-value is *p* = 0.006.

In female fetuses, prenatal hypoxia had no effect on fetal weight or abdominal girth but increased placental weight, which was significantly reduced by nMitoQ treatment (Table 3). nMitoQ treatment had no effect on female fetal weight or abdominal girth (Table 3). Prenatal hypoxia reduced placental efficiency and expressed as ratio of fetal weight/placental weight in female fetuses, which was not significantly improved by nMitoQ treatment; however, placental efficiency in the hypoxia/nMitoQ group was no longer significantly different normoxia/saline controls (Table 3). Crown-to-rump length was similar between all experimental groups in female fetuses (Table 3).

**Table 3:** Fetal and placental characteristics of female offspring.

Variables	Normoxia		p-Hypoxia		Main effect		
	Saline	nMitoQ	Saline	nMitoQ	p-Hypoxia	nMitoQ	Interaction
Fetal weight (g)	5.16 ± 0.29	5.81 ± 0.14	5.17 ± 0.16	5.26 ± 0.09	–	–	–
Placental weight (g)	0.56 ± 0.01	0.59 ± 0.01	0.62 ± 0.02	**0.56** ± **0.01** ^†^	–	–	*
Fetal weight/placental weight	9.06 ± 0.54	9.7 ± 0.31	7.7 ± 0.62	8.72 ± 0.44	–	–	–
Abdominal girth (cm)	3.67 ± 0.11	3.77 ± 0.13	3.38 ± 0.13	3.7 ± 0.12	–	–	–
Crown-rump length (cm)	4.7 ± 0.15	4.8 ± 0.10	4.56 ± 0.14	4.63 ± 0.07	–	–	–

Body weight, placental weight, placental efficiency (body weight/placental weight), abdominal girth, and crown-rump length from female fetuses collected on GD21. Data are represented as mean ± SEM. All groups were compared using a two-way ANOVA followed by Sidak’s *post hoc* test.

* *p* < 0.05 overall group effect of prenatal hypoxia.

† *p* < 0.05 versus corresponding hypoxia-saline group. Exact p-value is *p* = 0.04

### nMitoQ Treatment Improved Placental Oxidative/Nitrosative Stress in Female Offspring

**Figure 1:**
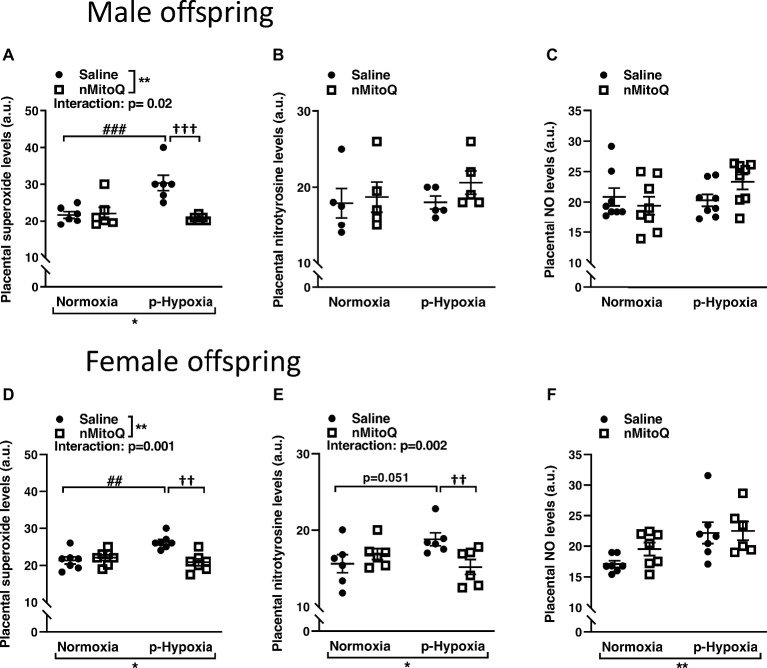
Effects of nMitoQ treatment on placental superoxide, peroxynitrite, and NO levels in normoxic and hypoxic placentae of both male and female offspring. Normoxic and hypoxic dams were treated with nMitoQ or saline, and superoxide levels were assessed by DHE staining in placentae from male **(A)** and female **(D)** fetuses; peroxynitrite levels were detected by staining for nitrotyrosine (the footprint of peroxynitrite production) in placentae from male **(B)** and female **(E)** fetuses, and NO levels were assessed by DAF-FM staining in placentae from male **(C)** and female **(F)** fetuses on GD21. Data are represented as mean ± SEM. a.u.: arbitrary units. All groups were compared using a two-way ANOVA followed by Sidak’s *post hoc* test (*n* = 6–7/group). *p < 0.05, **p < 0.01 group effect of prenatal hypoxia or nMitoQ treatment; ^##^*p* < 0.01, ^###^*p* < 0.001 compared to normoxia-saline; ^††^*p* < 0.01, ^†††^*p* < 0.001 compared to hypoxia-saline group.

Superoxide and peroxynitrite levels were assessed as markers of oxidative/nitrosative stress. In male offspring, superoxide levels were significantly increased in offspring exposed to prenatal hypoxia compared to normoxic control offspring (Figure 1A). nMitoQ treatment significantly decreased placental superoxide generation in prenatally hypoxic male offspring and had no effect in the control group (Figure 1A). Nitrotyrosine levels (Figure 1B) or nitric oxide levels (Figure 1C) were not affected by prenatal hypoxia or nMitoQ treatment in placentae from male offspring.

In female offspring, superoxide levels significantly increased in offspring exposed to prenatal hypoxia were significantly decreased by nMitoQ treatment in prenatally hypoxic offspring and had no effect in the control group (Figure 1D). Nitrotyrosine levels tended to be increased in the placentae of prenatally hypoxic female offspring (Figure 1E). Moreover, there was a significant interaction between nMitoQ treatment and prenatal hypoxia exposure in which nMitoQ treatment decreased nitrotyrosine levels in placentae of only female hypoxic offspring (Figure 1E). However, hypoxia significantly increased nitric oxide levels in placentae from female offspring, but there was no effect of nMitoQ treatment (Figure 1F).

### nMitoQ Treatment Decreased Markers of Placental and Fetal Hypoxia in Female Offspring

**Figure 2:**
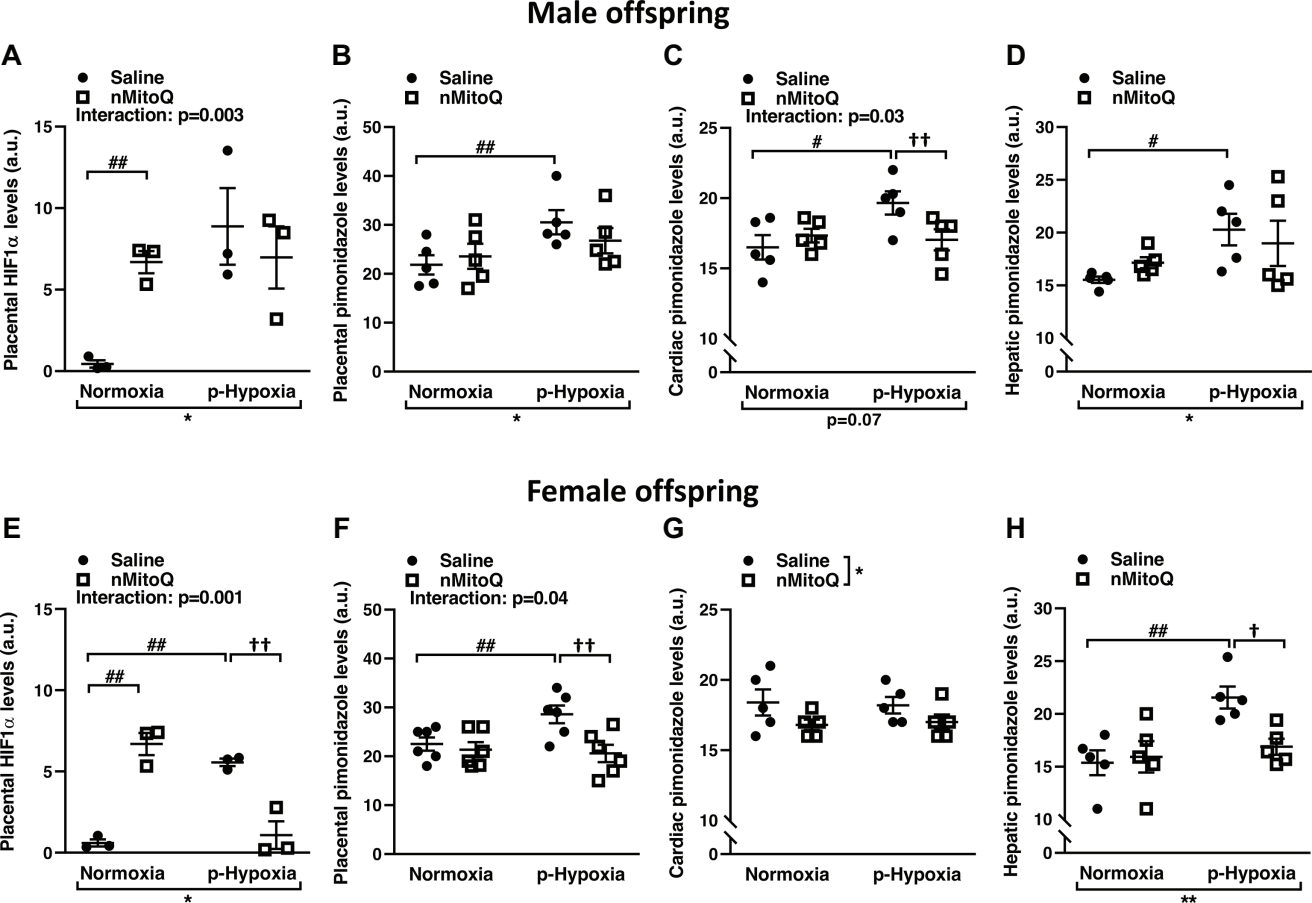
Effects of nMitoQ treatment on placental and fetal hypoxia. Expression of HIF1-α protein, a marker for tissue hypoxia in placentae obtained from male **(A)** and female **(E)** fetuses on GD21. Oxygenation levels as assessed by pimonidazole staining in the placentae of male **(B)** and female **(F)**, the cardiac tissues of male (**C)** and female **(G)** and hepatic tissues of male **(D)** and female **(H)** fetuses on GD21. Data are represented as mean ± SEM. a.u.: arbitrary units. All groups were compared using a two-way ANOVA followed by Sidak’s *post hoc* test (*n* = 5–6/group). *p < 0.05, **p < 0.01 group effect of prenatal hypoxia and nMitoQ treatment; ^#^*p* < 0.05, ^##^*p* < 0.01 compared to normoxia-saline; ^†^*p* < 0.05, ^††^*p* < 0.01 compared to hypoxia-saline group.

We next assessed HIF1-α protein levels as a marker for tissue hypoxia and confirmed placental oxygenation levels by pimonidazole staining. HIF1-α expression was significantly increased in prenatal hypoxia-exposed placentas from male offspring, which was not reduced by nMitoQ treatment (Figure 2A). There was a significant interaction between nMitoQ treatment and prenatal hypoxia in male placentae, whereby nMitoQ increased HIF1-α expression in placentae from normoxic male offspring (Figure 2A). Placental oxygenation was decreased (as shown by increased pimonidazole staining) in placentae of male offspring, which was unaffected by nMitoQ treatment (Figure 2B). Prenatal hypoxia decreased oxygenation in fetal hearts of only male offspring, while nMitoQ increased oxygenation in only prenatally hypoxic hearts of male offspring (Figure 2C). Hepatic oxygenation was reduced in livers of male offspring, but nMitoQ treatment had no effect (Figure 2D).

Prenatal hypoxia significantly increased placental HIF1-α expression in female offspring (Figure 2E). nMitoQ treatment decreased HIF1-α expression in placentae from female offspring exposed to prenatal hypoxia (Figure 2E). There was a significant interaction between nMitoQ treatment and prenatal hypoxia in female placentae, whereby nMitoQ increased HIF1-α expression in placentae from normoxic female offspring (Figure 2E). Placental oxygenation was also decreased (as shown by increased pimonidazole staining) in placentae of female offspring exposed to prenatal hypoxia (Figure 2F). nMitoQ treatment significantly increased oxygenation in placenta of only female offspring exposed to prenatal hypoxia (Figure 2F). There was no effect of prenatal hypoxia on oxygenation in fetal hearts of female offspring, while nMitoQ increased oxygenation in hearts of female offspring (Figure 2G). Hepatic oxygenation was reduced in livers of prenatally hypoxic female offspring, and nMitoQ treatment increased oxygenation in livers of only female offspring exposed to prenatal hypoxia (Figure 2H).

### nMitoQ Treatment Increased Angiogenesis and Vascularization in Placentae of Female Offspring

**Figure 3:**
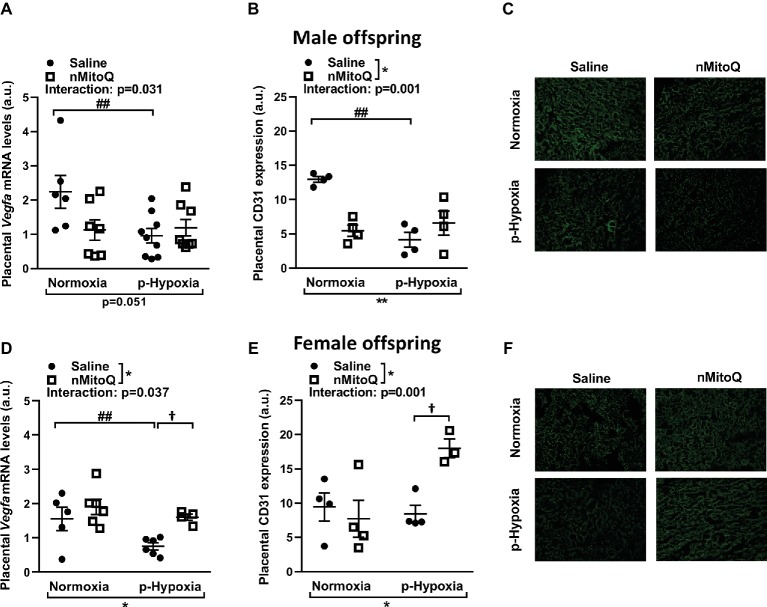
Effects of nMitoQ treatment on markers of angiogenesis and vascularization in prenatally hypoxic placentae of both male female offspring. Proangiogenic factor *Vegfa* mRNA levels were assessed by qPCR in placental tissue obtained from male **(A)** and female **(D)** fetuses on GD21. Feto-placental vascular capillaries as assessed by CD31 staining in placentae obtained from male **(B)** and female **(E)** fetuses. Representative images of CD31 stained placental labyrinth sections in placentae of male **(C)** and female offspring **(F)**. Data are represented as mean ± SEM. a.u.: arbitrary units. All groups were compared using a two-way ANOVA followed by Sidak’s *post hoc* test (*n* = 5–9/group). *p < 0.05, **p < 0.01 group effect of prenatal hypoxia and nMitoQ treatment; ^##^*p* < 0.01 compared to normoxia-saline; ^†^*p* < 0.05 compared to hypoxia-saline group.

Placental hypoxia is commonly associated with altered expression of the placental pro-angiogenic peptide vascular endothelial growth factor (VEGF; Lyall et al., 1997; Roh et al., 2005). In male offspring, prenatal hypoxia decreased placental *Vegf* mRNA expression, which was not altered by nMitoQ treatment (Figure 3A). Prenatal hypoxia also reduced CD31-positive area of staining (i.e., the fetal capillaries; Figures 3B,C). There was a significant interaction between nMitoQ treatment and prenatal hypoxia in male placentae, whereby nMitoQ reduced both *Vegf* and CD31 staining in placentae from normoxic male offspring (Figures 3A,B).

In female offspring, prenatal hypoxia decreased placental *Vegf* mRNA expression, which was increased by nMitoQ treatment in placentae from prenatally hypoxic female offspring (Figure 3D). CD31-positive area of staining (i.e., the fetal capillaries) was reduced by prenatal hypoxia in placentae from female offspring (Figures 3E,F). Similar to the *Vegf* expression pattern, there was a significant interaction between nMitoQ treatment and prenatal hypoxia in female placentae, whereby nMitoQ increased CD31 staining only in the placentae from prenatal hypoxic female offspring (Figure 3E).

**Figure 4:**
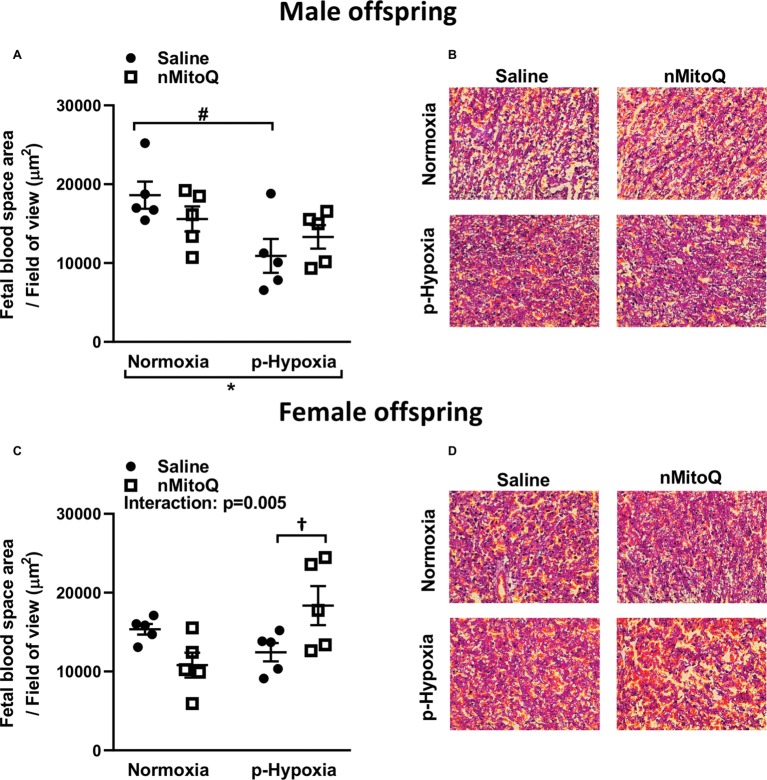
Effects of nMitoQ treatment on fetal blood space area in prenatally hypoxic placentae of both male and female offspring. Fetal blood space area per field of view in labyrinth zones of placenta obtained from male **(A)** and female **(C)** fetuses on GD21. Representative images of H&E stained placental labyrinth sections in placentae of males **(B)** and females **(D)**. Data are represented as mean ± SEM. All groups were compared using a two-way ANOVA followed by Sidak’s *post hoc* test (*n* = 5/group). *p < 0.05 group effect of prenatal hypoxia, ^#^*p* < 0.05 compared to normoxia-saline, ^†^*p* < 0.05 compared to hypoxia-saline group.

Sufficient fetal blood space in the placental labyrinth zone is essential for oxygen and nutrient exchange between the maternal and fetal circulations (Burton and Fowden, 2015). We found that prenatal hypoxia reduced fetal and maternal blood space area in placentae from only male offspring (Figures 4A,B). nMitoQ did not change fetal and maternal blood space area in placentae from males (Figure 4A); however, in female offspring, nMitoQ treatment increased fetal blood space area in placentae exposed to prenatal hypoxia (Figures 4C,D).

### nMitoQ Treatment Increased Placental Igf2 in Female Offspring

**Figure 5:**
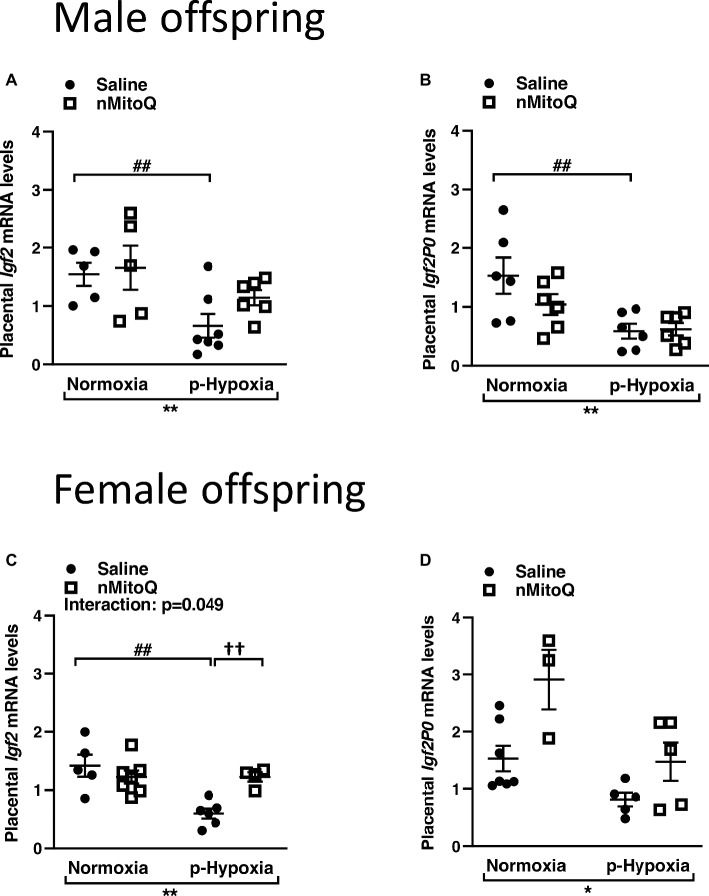
Effects of nMitoQ on placental *Igf2* mRNA expression in prenatally hypoxic placentae of both male and female offspring. Growth factor *Igf2* **(A,C)** and *Igf2p0* **(B,D)** mRNA levels as assessed by qPCR in placentae obtained from male **(A,B)** and female **(C,D)** fetuses on GD21. Data are represented as mean ± SEM. All groups were compared using a two-way ANOVA followed by Sidak’s *post hoc* test (*n* = 5–9/group). *p < 0.05, **p < 0.01 group effect of prenatal hypoxia; ^##^*p* < 0.01 compared to normoxia-saline; ^††^*p* < 0.01 compared to hypoxia-saline group.

**Figure 6:**
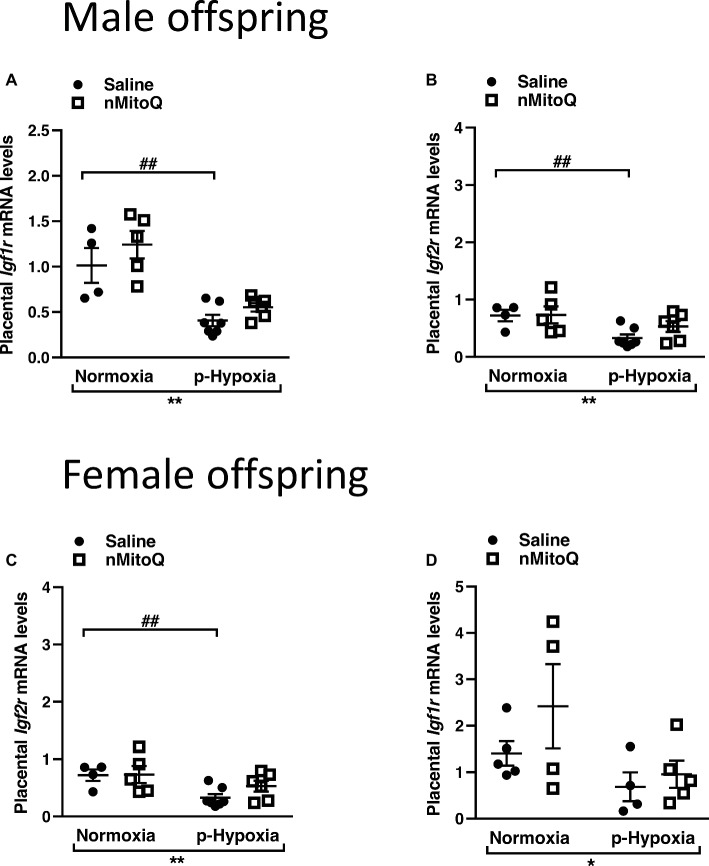
Effects of nMitoQ treatment on *Igf1r* and *Igf2r* mRNA expression in prenatally hypoxic placentae of both male and female offspring. Receptors for IGF2, *Igf1r* **(A,C)** and *Igf2r* **(B,D)** mRNA levels were assessed by qPCR in the labyrinth zones of placentae taken from male **(A,B)** and female **(C,D)** fetuses on GD21. Data are represented as mean ± SEM. All groups were compared using a two-way ANOVA followed by Sidak’s *post hoc* test (*n* = 4/group). *p < 0.05, **p < 0.01 group effect of prenatal hypoxia or nMitoQ treatment; ^##^ *p* < 0.01 compared to normoxia-saline.

IGF2 plays an important role in placental development (Fowden et al., 2006), and total placental *Igf2* and *Igf2* expressed only in the placental labyrinth region (i.e., *Igf2P0*) were shown to regulate the nutrient exchange characteristics of the placenta (Sibley et al., 2004). In male offspring, prenatal hypoxia decreased total *Igf2* and placental-specific *Igf2* expression, which was not affected by nMitoQ treatment (Figures 5A,B). In placentae from female offspring, total *Igf2* and placental-specific *Igf2* expression were significantly decreased by prenatal hypoxia (Figures 5C,D). Moreover, nMitoQ treatment increased *Igf2* expression in the prenatal hypoxia-exposed female placentae only (Figure 5C). Expression levels of the IGF2 receptors, *Igf1r* and *Igf2r*, were decreased in placentae of both prenatally hypoxic male and female offspring but were not affected by nMitoQ treatment (Figures 6A–D).

## Discussion

In the current study, we hypothesized the sex-specific effects of preventing placental oxidative/nitrosative stress using nMitoQ, with a specific focus on placental morphology. This is of particular interest because oxidative stress in the placenta has been implicated to play a key role in the etiology of pregnancy complications such as fetal hypoxia and IUGR. Hence, our study was designed to elucidate possible mechanism(s) in the placenta that may account for differences in placental and fetal oxygenation and weights following a hypoxic insult and the sexually dimorphic effects of nMitoQ treatment. We showed that a reduction in placental ROS/RNS by nMitoQ treatment improved oxygenation, increased expression of VEGFA (known to affect placental morphology), and increased expression of *Igf2*. Moreover, nMitoQ treatment was more effective in placentae of female offspring than males. Our data demonstrated that the placental response to prenatal hypoxia is different in males versus females and that within the same dams, the response of the placentae to nMitoQ treatment is sexually dimorphic, resulting in sex differences in the effect of nMitoQ treatment on placental oxidative stress, vascularization, and placental and fetal growth.

Given that oxidative stress is a central mediator of placental dysfunction, we and others have studied the use of an antioxidant to protect the placenta against oxidative stress (Phillips et al., 2017; Nuzzo et al., 2018). Recently, the antioxidant benefits of MitoQ treatment on placental adaptations from hypoxic pregnancies were extensively studied in male offspring (Nuzzo et al., 2018). Nuzzo et al. showed that MitoQ given daily to hypoxic pregnant dams in drinking water from GD6 to GD20 restored placental efficiency to control levels, increased absolute placental volume, fetal capillary surface area in placental labyrinth zone, and maternal blood spaces related to placentae from normoxic pregnancies (Nuzzo et al., 2018). The placental morphological adaptations (i.e., increased placental weight, increased fetal capillary surface area) to early-onset hypoxia (GD6–GD20) demonstrated in males were contrary to the placental morphological phenotype (i.e., unchanged placental weight and reduced fetal capillary area) observed in our model of late-onset hypoxia (GD15 –GD21) in male offspring. Thus, diverse prenatal hypoxia insults can affect pregnancies differently, which need to be taken into consideration for clinical translation of findings. In our study, using MitoQ encapsulated in nanoparticles (nMitoQ) to target the placenta in order to limit direct drug exposure to the fetus and avoid potential off-target effects during fetal development, we also observed beneficial effects; however, nMitoQ treatment was more effective in placentae of female offspring than males.

In our model of prenatal hypoxia, placental efficiency (fetal weight/placental weight) was reduced in both male and female fetuses, which was partially improved by nMitoQ treatment in both males and females. Interestingly, fetal weight was reduced by prenatal hypoxia only in males but not females, indicating that males may be more severely affected by prenatal hypoxia than females, as has been suggested in other studies (Stark et al., 2011; Muralimanoharan et al., 2015; Evans and Myatt, 2017). This reduction in fetal weight suggests insufficient oxygen and nutrient delivery to the fetus in the hypoxia exposed dams. Interestingly, in females, prenatal hypoxia increased placental weight, which was prevented by nMitoQ treatment. This may suggest that females, in compensation for the hypoxic environment, increased placental growth in order to increase oxygen and nutrient supply to the fetus and prevent growth restriction, which was indeed successful, as fetal weights in the females were not affected by prenatal hypoxia.

We showed that prenatal hypoxia increased oxidative stress (superoxide generation in male and female placentae), as demonstrated previously (Richter et al., 2012; Aljunaidy et al., 2018; Nuzzo et al., 2018), and nitrosative stress (peroxynitrite formation in only female placentae). Oxidative and nitrosative stresses can inhibit normal placental development in various ways (e.g., inhibit mitochondrial electron transport and oxidation of DNA) and potentially reduce oxygen and nutrient delivery, thus impairing fetal development (Droge, 2002; Thompson and Al-Hasan, 2012). The nMitoQ treatment was therefore specifically designed to prevent oxidative stress in the placenta, and indeed, nMitoQ treatment significantly decreased placental superoxide and peroxynitrite levels. This supports our hypothesis that using MitoQ loaded nanoparticles as a targeted delivery system to the placenta is an effective treatment against the generation of reactive oxygen/nitrogen species in the placenta in compromised pregnancies. Interestingly, in female offspring, we observed that the increased superoxide/peroxynitrite levels by prenatal hypoxia coincided with increased NO levels. Under normal pregnancy conditions, NO bioavailability promotes feto-placental vasodilation and induces angiogenesis (Cooke, 2003; Kwon et al., 2004). The placental circulation lacks autonomic and cholinergic innervation, thus feto-placental angiogenesis and placental vascular resistance and function are dependent on locally derived vasoactive factors (e.g., NO) and growth factors (e.g., VEGF and IGF2; Pacher et al., 2007; Krause et al., 2011; Kulandavelu et al., 2013). In the human placenta, all nitric oxide synthase (NOS) isoforms are expressed but differentially within the tissues. Endothelial NOS (eNOS) isoform is predominantly expressed in syncytiotrophoblasts and endothelial cells (reviewed by Aban et al., 2018), while neuronal NOS (nNOS) and inducible NOS (iNOS) isoforms are expressed in the placental smooth muscle cells, syncytiotrophoblasts, extravillous trophoblasts, and hofbauer cells of the villous stroma (reviewed by Krause et al., 2011). Moreover, studies have correlated increased placental vascular resistance and placental dysfunction to decreased eNOS and increased inducible NOS (iNOS) isoform expression, leading to increased nitrosative stress (Casanello and Sobrevia, 2002; Giannubilo et al., 2008). Interestingly, IUGR has been associated with higher levels of placental NO, together with increased nitrosative stress and inadequate feto-placental vascularization (Lyall et al., 1996; Tikvica et al., 2008; Myatt, 2010). Therefore, the increased NO levels in female placentae may be an immediate adaptive mechanism to prenatal hypoxia, which may be associated with placental vascular dysfunction. Although we have not assessed NOS isoforms and the contribution of various cell types to the increased placental NO levels in females, the above studies may suggest that increased iNOS expression and/or activity could be a potential source for increased placental NO observed in our model of prenatal hypoxia.

We found that prenatal hypoxia decreased placental and fetal oxygenation (in males and females), which coincided with an increase in HIF-1α levels in both sexes. Systemic and placental responses to hypoxia are orchestrated by hypoxia-inducible factors (such as HIF-1α), and being a marker of hypoxia, this finding is in accordance with the reduced placental oxygenation we observed. Notably, in only the female prenatally hypoxic placentae, nMitoQ treatment effectively improved placental oxygenation. Moreover, the effect of prenatal hypoxia on oxygenation was more pronounced in the female fetal liver than the fetal heart, as previously reported by our laboratory, but only in male offspring (Rueda-Clausen et al., 2011). Interestingly, nMitoQ treatment improved oxygenation in the hearts of both male and female fetuses, but in the liver, only female fetuses showed improved oxygenation with nMitoQ. Moreover, placental HIF-1α was only decreased by nMitoQ in the female placentae. This may explain why the female fetuses did not show any signs of significant growth restriction, as nMitoQ effectively decreased oxidative stress and HIF-1α and reduced placental weight in the female placentae. The underlying mechanisms for reduced effectiveness of nMitoQ treatment in males are not fully understood and remain to be further studied. A possible explanation might be that males show greater growth rate *in utero* (Parker et al., 1984); therefore, the higher oxygen and nutrient demand by the male fetus may predispose them to a greater risk of adverse developmental outcomes following the oxygen deprivation (Ozaki et al., 2001).

Oxidative stress is known to contribute to abnormal placental growth, function, and angiogenesis (Burton et al., 2009). As mentioned above, the placenta lacks autonomic innervation, and locally derived NO and growth factors such as the pro-angiogenic factor VEGF play essential roles in placental vascular development and function (Kulandavelu et al., 2013). HIF-1α (in hypoxic conditions) has been shown to increase VEGFA expression, while a reduction in VEGF has been observed in placentae from complicated pregnancies such as preeclampsia (Andraweera et al., 2012; Abe et al., 2013). Therefore, placental angiogenesis is dependent on locally derived VEGFA. Our study showed that prenatal hypoxia decreased *Vegfa* mRNA expression and the area of fetal blood capillaries (measured by endothelial cell marker CD31) in placentae of both male and female offspring, suggesting decreased angiogenesis and vascularization. The decreased *Vegfa* mRNA expression could be due to decreased binding of HIF-1α to the hypoxia responsive element (HRE) on the *Vegfa* gene, which was previously shown by Myatt et al. to decrease *Vegfa* expression in preeclamptic placentae with increased ROS (Muralimanoharan et al., 2012). Interestingly, nMitoQ treatment only improved *Vegfa* mRNA expression, the area of fetal blood capillaries, and the placental blood space in the female placentae but not in the male placentae. The specific mechanisms remain to be investigated, but it may be speculated that this could be mediated in part by increased placental NO levels present in the prenatally hypoxic placentae, which were not affected by nMitoQ treatment and could increase VEGFA. However, the effects of NO on VEGFA expression vary for different tissues and cell types (Abe et al., 2013); therefore, NO mediated VEGFA expression within the placenta may be highly dependent on the different cell types. Our data are in accordance with the previous studies, where MitoQ (not bound to nanoparticles) was shown to increase maternal blood space surface area in the placenta in males only (Nuzzo et al., 2018). Taken together, our data could suggest that reduction of superoxide by nMitoQ could lead to enhanced angiogenesis *via* increased *Vegfa* expression and fetal capillary area and blood space in placentae of female offspring.

Another important growth factor for placental morphogenesis is IGF2 (Sferruzzi-Perri et al., 2017). In humans, decreased placental IGF2 has been associated with IUGR (Diplas et al., 2009). In mice, genetic knockouts of *Igf2* showed impaired placental development and fetal growth with reduced placental transfer of nutrients to the fetus (Constancia et al., 2005; Coan et al., 2008). We observed that prenatal hypoxia decreased expression of *Igf2* and *Igf2P0* in the labyrinth zone of both male and female offspring. This is in line with the previous studies showing that maternal exposure to hypoxia has direct effects on total *Igf2* expression in the placenta: maternal exposure to 10–12% oxygen during late gestation decreased *Igf2* expression, which affected the labyrinth layer morphology and decreased maternal blood space (Cuffe et al., 2014; Higgins et al., 2016). Hence, the reduction in placental *Igf2* expression could also explain the decreased fetal blood spaces by prenatal hypoxia that we observed. The expression of *Igf2* is regulated by methylation of the *Igf2* gene at the imprinting control region (ICR) and the differentially methylated region 2 (DMR2). Recent studies suggest that a suboptimal intrauterine environment leads to epigenetic changes in the *Igf2* gene and associated *Igf2* expression in growth restricted offspring of rats exposed to bilateral uterine artery ligation (Gonzalez-Rodriguez et al., 2016). Interestingly, hypoxic stress responses in general appear to be driven by epigenetic changes (Cerda and Weitzman, 1997). For example, prenatal hypoxia increased placental ROS and DNA methylation enzymes (Gheorghe et al., 2007). Therefore, we speculate that the methylation status at ICR and DMR2 may differ in a sex-specific manner, and changes in DNA methylation could account for the altered *Igf2* expression, which will be the focus for our future investigations.

It is well known that the placenta functions and adapts to an adverse intrauterine environment in a sex-specific manner (reviewed by Clifton, 2010). Furthermore, generation of oxidative stress differs in both male and female fetuses and placentae under conditions of adverse prenatal stress (Chaudhari et al., 2014). Our current study further demonstrates a dichotomous sex-specific and nMitoQ-specific effect on the placenta and ultimately fetal development. Overall, our data suggested that nMitoQ treatment decreased oxidative/nitrosative stress, improved oxygenation, was effective in bringing *Vegfa* and *Igf2* expression back to control levels, and increased fetal blood space only in female placentae, thereby showing that nMitoQ treatment may protect offspring from the detrimental effects of a hypoxic insult in a sexually dimorphic manner with increased effectiveness in females. Of note, our data showed that nMitoQ treatment also affected the placenta in the control groups. For example, in normoxic controls, nMitoQ treatment increased HIF-1α expression in both male and female placentae and reduced CD31 area in the male offspring. Previous studies have demonstrated that maternal antioxidant supplementation with ascorbic acid in normal pregnancies was associated with vascular dysfunction and weight gain (Richter et al., 2012). Therefore, continuing these studies we need to keep in mind that this treatment is specifically designed to treat pregnancies complicated by fetal hypoxia and be aware that there might be detrimental effects of maternal antioxidant intervention during normal pregnancy.

To conclude, we demonstrated that nMitoQ treatment reduced placental nitrosative stress and improved oxygenation and placental morphology *via* increased VEGFA and IGF2 expression in a sex-specific manner, showing more effectiveness in placentae from female offspring. Moreover, our study shows that male fetuses appear to be more susceptible to an adverse *in utero* environment. Although the exact mechanism(s) remain to be further investigated, a higher pro-oxidant state with reduced antioxidant capacity in the male placentae may explain the increased susceptibility of the male offspring under adverse conditions. In addition, our current study illustrates that the placenta is a contributing factor in the sexual dimorphism that has been observed in fetal programming. Thus, sex differences will need to be taken into account when developing placental-targeted therapeutic interventions to prevent fetal hypoxia and ultimately optimize fetal development in complicated pregnancies.

## Data Availability

The datasets generated for this study are available on request to the corresponding author.

## Ethics Statement

All procedures described were approved by the University of Alberta Animal Policy and Welfare Committee and were in accordance with the guidelines of the Canadian Council on Animal Care (AUP #242).

## Author Contributions

EG, MA, FS, JM, TP, CPC, C-LC, and SD contributed to experimental design. EG, MA, and RK participated in data acquisition. EG, MA, FS, JM, C-LC, and SD analyzed the data. EG, FS, C-LC, and SD were involved in drafting of the manuscript. EG, MA, FS, JM, TP, CPC, C-LC, and SD revised the final version of the manuscript. TP and CPC provided the study materials.

### Conflict of Interest Statement

The University of Bristol (TP and CC) has submitted a patent application for the nanoparticle formulation used in this study and its application to preeclampsia and related diseases.

The remaining authors declare that the research was conducted in the absence of any commercial or financial relationships that could be construed as a potential conflict of interest.
